# Xeroderma Pigmentosum: Low Prevalence of Germline *XPA* Mutations in a Brazilian XP Population

**DOI:** 10.3390/ijms16048988

**Published:** 2015-04-22

**Authors:** Karina Miranda Santiago, Amanda França de Nóbrega, Rafael Malagoli Rocha, Silvia Regina Rogatto, Maria Isabel Achatz

**Affiliations:** 1 International Research Center, A.C. Camargo Cancer Center, São Paulo, SP 01508-010, Brazil; E-Mail: karina.santiago@accamargo.org.br; 2 Department of Oncogenetics, A.C. Camargo Cancer Center, São Paulo, SP 01509-900, Brazil; E-Mail: amanda.nobrega@accamargo.org.br; 3 Molecular Morphology Group, Investigative Pathology Department, A.C. Camargo Cancer Center, São Paulo, SP 01509-900, Brazil; E-Mail: rafael.malagoli@gmail.com; 4 International Research Center, A.C. Camargo Cancer Center, National Institute of Science and Technology in Oncogenomics (INCITO), São Paulo, SP 01508-010, Brazil; 5 Department of Urology, Faculty of Medicine, University of São Paulo State, Botucatu, SP 18618-970, Brazil

**Keywords:** xeroderma pigmentosum syndrome, *XPA* gene, skin cancer, neurodegeneration

## Abstract

Xeroderma pigmentosum (XP) is a rare autosomal recessive disorder characterized by DNA repair defects that cause photophobia, sunlight-induced cancers, and neurodegeneration. Prevalence of germline mutations in the nucleotide excision repair gene *XPA* vary significantly in different populations. No Brazilian patients have been reported to carry a germline mutation in this gene. In this study, the germline mutational status of *XPA* was determined in Brazilian patients exhibiting major clinical features of XP syndrome. The study was conducted on 27 unrelated patients from select Brazilian families. A biallelic inactivating transition mutation c.619C>T (p.Arg207Ter) was identified in only one patient with a history of neurological impairment and mild skin abnormalities. These findings suggest that XP syndrome is rarely associated with inherited disease-causing *XPA* mutations in the Brazilian population. Additionally, this report demonstrates the effectiveness of genotype-phenotype correlation as a valuable tool to guide direct genetic screening.

## 1. Introduction

Xeroderma pigmentosum (XP) is a rare autosomal recessive syndrome associated with mutations in one of the seven genes involved in Nucleotide Excision Repair pathway of damaged DNA. Patients with XP variant type have defective translesion synthesis due the inactivation of *POLH*. Based on which gene is affected, patients are classified into XP complementation groups (XP-A to XP-G and X-PV) [1,2]. The reduced capacity for removing bulky DNA lesions and the failure in bypassing DNA alterations in an error-free manner during cellular replication increase the risk of developing UV-induced skin and mucous membrane lesions.

The first clinical manifestations of XP occur early in life and may include cutaneous and ophthalmic photosensitivity related to sun exposure. XP patients have a 10,000-fold increased risk of developing non-melanoma skin cancer before the age of 10 and a 2000-fold increase in the incidence of malignant melanoma before the age of 20 compared to the general population. Neurological degeneration is observed in 20%–30% of all patients with XP but mainly affects carriers of *XPA* or *XPD* mutations [1,2,3,4].

The prevalence of germline *XPA* mutations in XP patients varies, accounting for 55% of all Japanese XP cases and 9% of United States XP patients [5,6,7,8,9]. Only a few studies involving a small number of Brazilian XP cases have been published; to the best of our knowledge, the frequency of germline *XPA* mutations in this population has not yet been described [10,11,12,13], thereby hindering the evaluation of genotype-phenotype correlations.

The main purpose of this study was to screen germline *XPA* pathogenic alterations in a group of Brazilian patients clinically diagnosed with XP syndrome. The findings suggest that XP syndrome is rarely associated with *XPA* mutations in the Brazilian population.

## 2. Results and Discussion

### 2.1. Germline Mutation Spectrum of XP Syndrome and Clinical Profile

Twenty-seven unrelated patients who fulfilled the main criteria for XP syndrome were evaluated for germline *XPA* mutations. The mean age of the patients at admission was 26.2 years (range, 1–73 years). All the subjects presented with classical progeroid skin abnormalities covering entirely sun-exposed areas: severe erythema, sunburn lentigines, telangiectasia, bullae, xerosis, skin atrophy, hypopigmented patches and actinic keratosis. The mean ages at presentation of first symptoms and first biopsied lesion were 2.7 and 9.4 years, respectively. Eleven unrelated patients had at least one family member affected by classical XP skin abnormalities.

The paradoxical associations between acute sunburn reactions and decreased risk of developing skin cancers [14] as well as between burning on minimal sun exposure and increased risk for neurological degeneration [15] are well-known genotype-phenotype correlations observed in XP-A patients [9,16]. Therefore, we assumed there was a high probability of finding *XPA* mutations in XP patients with neurologic impairment. Indeed, a germline *XPA* mutation was detected in one patient presenting with such a phenotype; a detailed description of this patient can be found in the Case Report section. Hence, the phenotype-genotype correlation provided relevant support for detecting mutations when an XP patient is referred for a genetic test in a setting where at least eight genes can potentially be mutated.

In addition to evaluating mutations in all the exons and intron-exon junctions, we also investigated a polymorphic sequence in the 5' noncoding region (c.-4A>G) to ensure that no technical issues were involved in the 26 negative patients. Ten patients were genotyped for the minor allele of rs1800975 *XPA*, thus excluding the possibility of sequencing failure. Although this polymorphism has been widely studied, its role on cancer is unclear [17].

**Figure 1:**
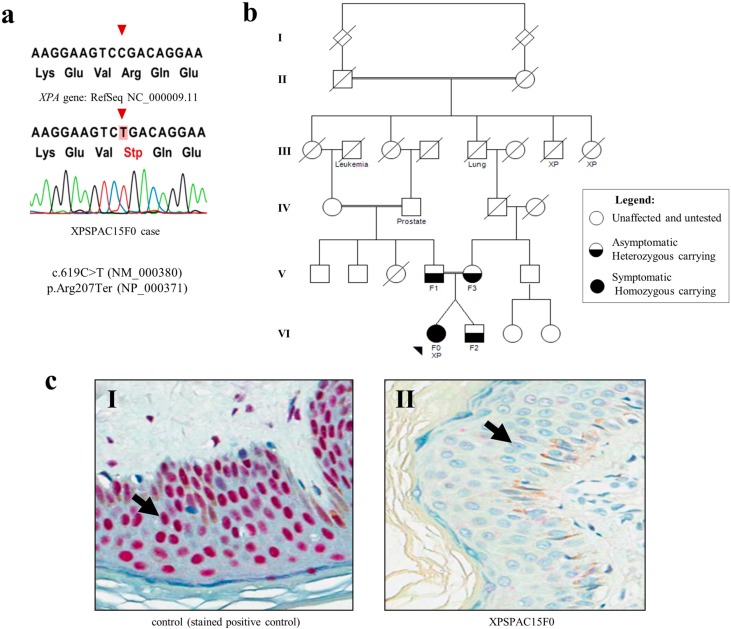
*XPA* pathogenic mutation and the absence of protein expression. (a) Electropherogram displaying the genomic DNA sequence for patient XPSPAC15F0: a C to T homozygous substitution was observed at *XPA* position 619 of transcript sequence NM_000380.3; (**b**) Segregation of the *XPA* mutation was achieved for the family of patient XPSPAC15F0 (VI: F0): her unaffected twin brother (VI: F2) and her parents (V: F1 and V: F3) are heterozygous carriers; (c) Immunohistochemistry revealed (**c**, **I**) the nuclear expression of XPA protein in a non-tumoral skin cells (the black arrow points to a representative cell) from a clinically non-xeroderma pigmentosum (XP) case unrelated to the family (positive control) and (**c**, **II**) the absence of XPA immunostaining in non-tumoral skin cells (black arrow depicts a representative cell) surrounding a basal cell carcinoma (BCC) from our patient with an *XPA* c.619C>T mutation. Selected photomicrographs were captured at 200× magnification.

The inactivating *XPA* mutation found in our patient was previously described in the cell line XP12RO [18,19,20]. This alteration refers to a biallelic transition at exon 5 c.619C>T that results in the stop codon p.Arg207Ter, which sits in the DNA binding region (Figure 1a). Consequently, impaired DNA damage recognition is expected. Segregation of this mutation was investigated in the asymptomatic patient’s relatives (second-cousin parents and unaffected brother) and revealed that her healthy dizygotic twin brother and both parents carried one copy of the variant (Figure 1b).

The complete absence of XPA protein expression due to this mutation was confirmed by immunohistochemistry (Figure 1c, II) for the *XPA*-positive patient, probably as a result of nonsense-mediated mRNA decay. In addition, XPA protein expression was evaluated in five of the 26 patients who tested negative for *XPA* mutations, and normal expression was observed.

Although the complete loss of protein production would suggest a severe form of the disease, the clinical presentation of the XP-A Brazilian patient was mild [6,21]. The direct correlation of sun exposition, skin phototype and habits in XP patients is well described in the literature. High parental awareness and effective sun protection may have prevented more severe cutaneous manifestations [2], and for this reason, the phenotype-genotype correlations are difficult to replicate in patients. Genetic counseling may provide education and stimulate patients to develop beneficial habits, thus reducing environmental effects and providing patient awareness.

Nonetheless, neurodegeneration appears to progress independently from UV-induced DNA lesions [22]. The brain is exposed to high levels of oxidative stress, and the accumulation of unrepaired endogenous oxidative DNA bulky lesions could contribute to generalized brain atrophy and associated symptoms [6,22,23,24,25,26].

In this study, we report one case of XP syndrome from complementation group A (XPSPAC15F0); the twin brother and parents of this patient were heterozygous for the *XPA* mutation. This study is the initial finding of a pioneering effort to generate a systematic clinical and molecular description of Brazilian patients with XP.

### 2.2. Case Report

**Figure 2:**
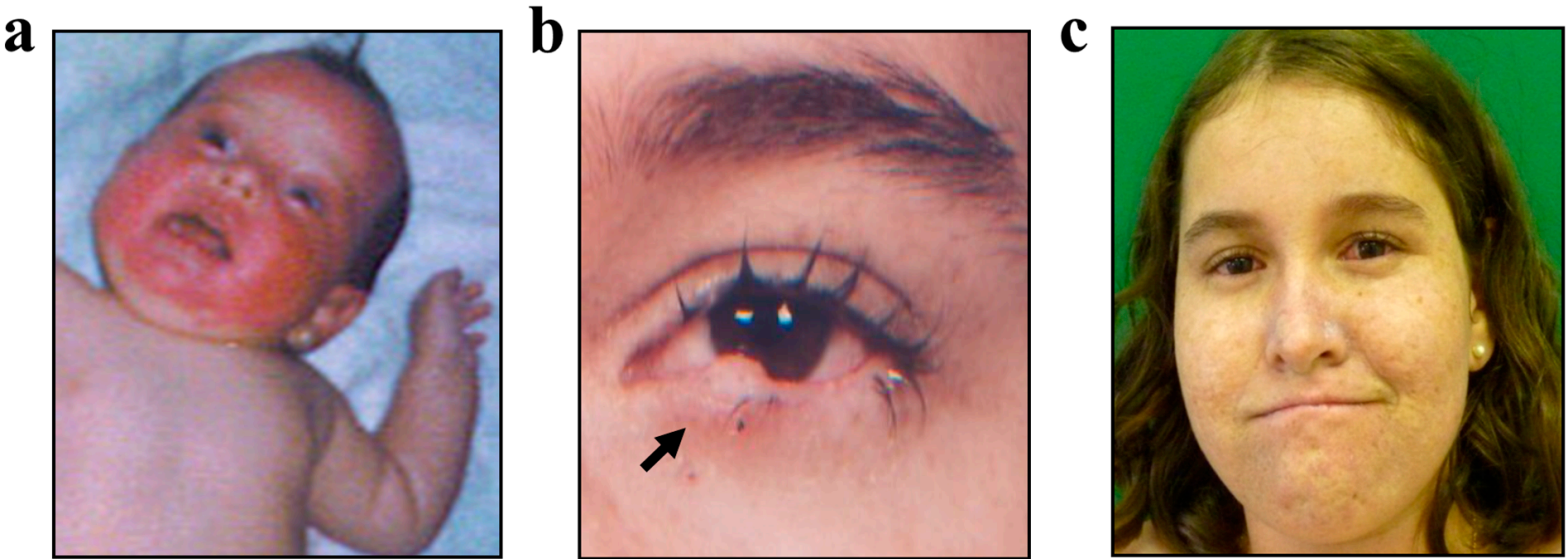
Skin aspects of XP patient. (**a**) Severe sunburn on face with blistering and erythema before the age of one month; (**b**) The biopsy of the lesion on the anterior lamella of 1.7 cm × 0.5 cm × 0.4 cm in size (black arrow) showed actinic keratosis and microcarcinoma invading the superficial dermis; (**c**) By the age of 11, cutaneous findings included disperse and moderate skin poikiloderma with hyper- and hypopigmentation in addition to telangiectasia of the ocular conjunctiva.

We report a 15-year-old female who presented with a disseminated skin rash at the age of one month as well as with acute burning and erythema on the face after minimal sun exposure (Figure 2a). At three months, she was diagnosed with sepsis.

These first symptoms resulted in an initial diagnosis of cutaneous porphyria. At the age of 4, she was diagnosed with XP syndrome after the first facial skin lesion appeared. The histopathological findings of this lesion revealed actinic keratosis. Progressively, hyperpigmentation on sun-exposed areas and intense photophobia became more apparent (Figure 2b). By 15 years old, she had 13 skin lesions removed mostly from the face, eyes, lips, neck, superior limbs and trunk. Six malignant lesions were diagnosed, including one squamous cell carcinoma, one basaloid squamous cell carcinoma, two basal cell carcinomas and two carcinomas of unspecific histology.

Signs of neurological impairment were initially noticed at age five. By age 11, added to a moderate cutaneous and ophthalmic photosensitivity (Figure 2c), mild cognitive and motor impairment, short stature (41.6 kg and 136 cm) and reduced head circumference (49 cm) were observed. A brain magnetic resonance imaging study was performed at 13 years old and revealed microcephaly, *ex-vacuo* ventriculomegaly due to parenchymal brain atrophy (commonly associated with aging), and discrete/diffuse bilateral/symmetric white matter hyperintensities on T2-weighted and FLAIR images (suggestive of demyelination or gliosis). Currently, at 15 years old, the patient has progressed to severe ataxia and is being followed by periodical clinical evaluation.

## 3. Experimental Section

### 3.1. Enrollment of Patients and Ethics Statement

This study was approved by the A.C. Camargo Cancer Center Ethics Committee (No. 1302/09), São Paulo, Brazil.

Twenty-seven unrelated XP patients were included. All of them were clinically diagnosed with XP syndrome by a dermatologist in childhood. At admission to the Department of Oncogenetics, patients presented with poikiloderma skin aspects and multiple pre-malignant or malignant skin lesions (keratoses, lentigines, nevi, basal cell carcinoma, squamous cell carcinoma and melanoma).

Individuals enrolled in this study provided written informed consent to publish health-related details.

### 3.2. Genomic DNA Extraction

Genomic DNA was isolated from 4 mL of peripheral blood collected in EDTA anticoagulant tubes. Samples were processed in a manner standardized by the A.C. Camargo Cancer Center Macromolecule Bank [27]. Initially, blood was processed by gradient centrifugation using 1× TE buffer, pH 8 (100 mM Tris and 10 mM EDTA), to isolate mononuclear blood cells. DNA was extracted using Gentra Puregene Blood Kit (Qiagen, Valencia, CA, USA) according to the manufacturer’s recommendations.

### 3.3. XPA Direct Sequencing

Direct bidirectional sequencing of the complete coding region was performed using six primer pairs for the *XPA* gene (Gene ID: 7507) (Table 1).

**Table 1:** Primers used to screen *XPA* mutations by direct sequencing.

Exon	Size (bp)	Forward Primer	Reverse Primer
1	483	AGGCGCTCTCACTCAGAAAG	GTGGACAGGACGCTTTGAC
2	523	AGACTAGCTGGGACCTTCAGT	AACAACAGAGAGCAGCAACC
3	419	GGCATTGCATACATGCTG	ACCATCGGCATCCTTCCTAT
4	645	CCTAGAGCCTTTTCCCTTGC	CCAGCCTGAGTGACAGAGTG
4	337	GCTGTGTGTGCCCCTAAGTTGC	AGCAAAAGCCAAACCAATTATGAC
5	611	GTGAGCCCACCACAGTTGAT	GGTTTGAGCTTAGTGCCTTG
7	574	CTCTTGTTTCACACTGCTCCAG	CCAGGTGACCTTCACTGAAAC
7	594	GTGAGGTAAGAAAGTAAGTTTGCCAAG	TCTAGCACTCAGCTCCCATCTCTG

bp: Base pair.

High quality genomic DNA (25 ng) was amplified using Platinum Taq DNA Polymerase High Fidelity (Invitrogen/Life Technologies, Carlsbad, CA, USA). Sanger sequencing was performed using BigDye Terminator v3.1 chemistry (Applied Biosystems/Life Technologies, Foster City, CA, USA) [28]. Data were generated on an ABI Prism 3130xl Genetic Analyzer (Applied Biosystems/Life Technologies) and were analyzed using the CLC Main Workbench 5.0.2. (CLC bio, Mühltal, Germany) with the human reference sequence GRCh37.p13 (HG19), RefSeq Assembly ID: NC_000009.11. The presence of mutations was confirmed by performing an additional reaction using DNA extracted from a second blood collection as well as by performing mutation segregation on the parents.

### 3.4. XPA Protein Expression by Immunohistochemistry

For the select patients with a mutation, immunohistochemistry was performed on 4-µm formalin-fixed, paraffin-embedded skin tumor sections with normal adjacent tissues. Tissue sections were processed and immunostained using Ventana Benchmark XT (Ventana Medical Systems, Tucson, AZ, USA) in combination with an ultraView Universal Alkaline Phosphatase Red Detection Kit (Ventana Medical Systems, Tucson, AZ, USA) according to the manufacturer’s recommendations. The mouse monoclonal anti-XPA IgG1 antibody clone 12F5 (1:100 dilution; Abcam, Cambridge, MA, USA) was used. Normal skin tissue was included as a positive control. The primary antibody was omitted in the negative control. ScanScope (Aperio Technologies, Vista, CA, USA) and the ScanScope Console v.10.2.0.2314/Controller v.10.2.0.2317 were used to capture the images at 200× magnification. The analysis was non-quantitative; the presence of staining was observed independent of staining intensity.

## 4. Conclusions

This study provides the first report of Brazilian XP patients, one of whom presented with an *XPA* mutation. These findings revealed that *XPA* mutations are not the major cause of XP syndrome in Brazil.
